# Activation of immune responses against the basement membrane component collagen type IV does not affect the development of atherosclerosis in ApoE-deficient mice

**DOI:** 10.1038/s41598-019-42375-8

**Published:** 2019-04-12

**Authors:** J. Vallejo, P. Dunér, F. To, D. Engelbertsen, I. Gonçalves, J. Nilsson, E. Bengtsson

**Affiliations:** Department of Clinical Sciences Malmö, Skåne University Hospital, Lund University, Malmö, Sweden

## Abstract

Oxidation of low-density lipoprotein (LDL) in the arterial extracellular matrix results in malondialdehyde (MDA)-modifications of surrounding matrix proteins. We have recently demonstrated an association between high levels of autoantibodies against MDA-modified collagen type IV and risk for development of myocardial infarction. Collagen type IV is an important component of the endothelial basement membrane and influences smooth muscle cell function. We hypothesized that immune responses against collagen type IV could contribute to vascular injury affecting the development of atherosclerosis. To investigate this possibility, we induced an antibody-response against collagen type IV in apolipoprotein E (Apo E)-deficient mice. Female ApoE^−/−^ mice on C57BL/6 background were immunized with α1α2 type IV collagen chain peptides linked to the immune-enhancer PADRE, PADRE alone or PBS at 12 weeks of age with three subsequent booster injections before the mice were killed at 23 weeks of age. Immunization of PADRE alone induced autoantibodies against PADRE, increased IL-4 secretion from splenocytes and reduced SMC content in the subvalvular plaques. Immunization with peptides of α1α2 type IV collagen chains induced a strong IgG1antibody response against collagen type IV peptides without affecting the distribution of T cell populations, plasma cytokine or lipid levels. There were no differences in atherosclerotic plaque development between collagen α1α2(IV)-PADRE immunized mice and control mice. Our findings demonstrate that the presence of antibodies against the basement membrane component collagen type IV does not affect atherosclerosis development in ApoE^−/−^ mice. This suggests that the association between autoantibodies against collagen type IV and risk for myocardial infarction found in humans does not reflect a pathogenic role of these autoantibodies.

## Introduction

Atherosclerosis is initiated by infiltration and accumulation of lipids in the vessel wall where these lipids bind to subendothelial extracellular matrix components. The subendothelial retention and oxidation of LDL may result in MDA-modifications of surrounding extracellular matrix proteins, inducing an immune response against the basement membrane^1^. Antibodies against MDA-modified apolipoprotein B100 or extracellular matrix proteins have previously been reported in patients^1–4^, and influence atherosclerotic lesion formation in mice^2,5,6^. In a previous study we found that MDA-collagen type IV was increased in carotid plaques from patients with cerebrovascular symptoms^7^. Furthermore, antibodies against MDA-collagen type IV were associated with increased risk for myocardial infarction^8^, indicating that immune responses against the basement membrane could be of importance for cardiovascular events.

Collagen type IV is one of the most abundant proteins in the basement membrane, which underlies endothelial cells and surrounds smooth muscle cells^9^. Collagen type IV confers stability through its formation of networks, as well as regulates cellular behavior. In mammals, collagen type IV contains six different alpha chains composed of an N-terminal 7S domain, a collagenous triple helical domain, and C-terminal non-collagenous globular domain^10^. Collagen type IV alpha 1 and alpha 2 chains (α1α2(IV)) are present in all tissues, whereas the other collagen IV chains have a more restricted distribution^9^. Collagen IV α1 has 83.5% homology between human and mouse species, whereas for collagen IV α2 it is even higher, reaching 90.6%^11,12^. *COL4A1* and *COL4A2* genes are encoded in α1(IV) and α2(IV), and the genes are located next to each other in the chromosome 13q34^13,14^. Genome-wide association studies have identified an association between coronary heart disease risk and SNP rs4773144, present in the *COL4A1-COL4A2* locus^15,16^. This SNP was shown to affect *COL4A1* and *COL4A2* expression, as well as atherosclerotic plaque stability^17^. Furthermore, mutations in these genes have been reported to cause hemorrhagic stroke in humans^18,19^.

Pan-DR epitopes (PADRE), a potent immunogenic 13 amino acid long peptide able to generate T helper responses in humans, is used as carrier in vaccines designed for human use^20,21^. It is utilized as an enhancer of antibody immune responses in different diseases, including atherosclerotic disease in LDLr^−/−^ mice^22^.

The aim of the current study was to investigate if immune responses against the basement membrane collagen type IV affect atherosclerotic plaques. For this purpose, antibodies against collagen type IV were induced in atherosclerotic ApoE^−/−^ mice using collagen α1α2(IV)-peptides coupled to PADRE (α1α2(IV)-PADRE) and atherosclerotic plaque formation was assessed.

## Material and Methods

### Proteins and peptides for immunization of mice

Mouse collagen type IV was purchased from Corning, MA, USA and MDA-modified as previously described^1^. Collagen type IV α1 (ATIERSEMFKKPTPSTLKAGELRTHVS) (α1(IV)), and collagen type IV α2 (TTIPEQNFQSTPSADTLKAGLIRTHIS) (α2(IV)) peptides^23^, were synthesized and coupled to PADRE (AKFVAAWTLKAAA) via a cysteine residue located in the C-terminal of PADRE (Biopeptide, San Diego, CA, USA).

### Mice

In initital experiments ApoE^−/−^ mice were immunized with 60 µg MDA-modified or native collagen type IV (without PADRE or mixed with PADRE), using Alum (aluminum hydroxide) (Thermo Scientific, Rockford, IL, USA), Freund’s incomplete (Sigma-Aldrich, St Louis, MO, USA) or Freund’s complete (Sigma-Aldrich, St Louis, MO, USA) as adjuvant. Since neither experiment yielded a consistent antibody response, in the next experiments we used collagen type IV α1 and α2 peptides^23^ conjugated to PADRE as antigens. Female ApoE^−/−^ mice on C57BL/6 background were purchased from Jackson Laboratories (Bar Harbor, ME, USA). Mice (n = 85) were immunized subcutaneously with collagen α1α2(IV)-PADRE (n = 20 for each concentration), PADRE alone (n = 15 for each concentration) or PBS (n = 15) at 12 weeks of age. Booster injections were performed at 14 and 17 weeks of age. A last booster was given one week before mice were euthanized. Each injection contained a mixture of the same amount of collagen α1(IV)-PADRE and α2(IV)-PADRE (25 μg or 50 μg per injection), PADRE (8 μg or 16 μg per injection) or PBS. Peptides or PBS were mixed with an equal volume of Alum (aluminum hydroxide) (Thermo Scientific, Rockford, IL, USA). The mice were fed a cholesterol diet (0.15% cholesterol, 21% fat; Lantmännen, Stockholm, Sweden) from the age of 12 weeks. At 23 weeks of age mice were anaesthetized by inhalation of 2.5% isoflurane (Isoba, Shering-Plough Animal health), killed by intraperitoneal injection of 300 μL of a solution consisting of 5 ml 0.9% NaCl, 1.1 ml 50 mg/ml ketamine (Ketaminol, Intervet, Stockholm, Sweden) and 0.9 ml 20 mg/ml xylazine (Rompun, Bayer, Leverkusen, Germany) followed by exsanguination by cardiac puncture and perfusion with 0.15 mol/L PBS, pH 7.4 (Sigma, St Louis, MO, USA). Upon termination the mice were weighed, and spleens, aortas and hearts were dissected. The aorta was dissected free of connective tissue and fat, cut longitudinally, and mounted *en face* lumen-side up on ovalbumin (Sigma-Aldrich, St Louis, MO, USA)-coated slides^24^ and stored in Histochoice (Amresco, Solon, Ohio, USA) fixative at 4 °C until processing. Blood was collected from cardiac punction, 0.5 M EDTA was added before centrifugation at 3000 g, and the plasma was stored at −80 °C until analysis. Hearts and brachiocephalic arteries were snap frozen in liquid nitrogen and stored at −80 °C until further processing. The study was carried out in accordance with the recommendations of the Guide for the Care and Use of Laboratory Animals of the National Institute of Health. All protocols were approved by the Malmö/Lund Animal Care and Use Committee.

### Isolation and culture of splenocytes

Spleens were harvested in ice-cold RPMI-1640 medium (Gibco, Stockholm, Sweden) and stored on ice until processing within hours. Spleens were pressed against a 70 um nylon cell strainer (Falcon, New York, NY, USA) to obtain a single cell suspension of splenocytes, followed by a washing step with RPMI. The pellets were resuspended and mixed in blood cell lysis buffer (Sigma-Aldrich, St Louis, MO, USA) to remove erythrocytes. Cells were counted and suspended in complete RPMI containing 1 mmol/L sodium pyruvate, 10 mmol/L Hepes, 50 U penicillin, 50 µg/mL streptomycin, 0.05 mmol/L 2-mercaptoethanol, 2 mmol/L L-glutamine and 10% heat inactivated fetal bovine serum (FBS) all purchased from Gibco (Stockholm, Sweden). One million splenocytes were cultured in microtiter 48-well plates and stimulated for 18 h with anti-CD3/CD28 beads. Cells were spun down at 1000 g for 5 min at 4 °C and supernatants were stored at −80 °C until further analysis.

### Flow cytometry

Half a million splenocytes were stained in each FACS tube. First, cells were washed with staining buffer (Biolegend, San Diego, CA, USA), and centrifuged at 800 g for 5 min at 4 °C. They were then stained with fluorochrome-conjugated antibodies after blocking of Fc receptors (CD16/32, Biolegend, San Diego, CA, USA) for 5 minutes. The following antibodies were used: FoxP3-PE, CD3-PE/Cy7, CD25-APC, CD4-PB, CD8a-FITC and CD3-PE-Cy7, all from Biolegend (San Diego, CA, USA). The cells were stained 30 min on ice in the dark with antibodies binding extracellular targets. Cells were then washed to remove unbound antibodies, incubated with Fixation/Permeabilization buffers (eBioscience, San Diego, CA, USA) for 30 min on ice and blocked with CD16/32 prior incubation with anti-FoxP3 for 30 min. Cells were resuspended in PBS buffer containing 1% BSA and 0.5 mM EDTA. Cells were run on Gallios flow cytometer (Beckman Coulter, High Wycombe, UK) and analyzed with FlowJo software V.10 (Tree Star, Inc. Ashland, OR, USA).

### T cell related cytokines and endothelial growth factors analysis

Interferon gamma (IFN-γ), interleukin (IL)-12, IL-4, IL-5, IL-6, IL-10, IL-13, and IL-17A in supernatants from splenocytes or from mouse plasma analyzed by multiplex technology using Luminex R&D Systems (Minneapolis, MN, USA) or Millipore Technology (Merck, Kenilworth, NJ, USA). Hepatocyte growth factor (HGF) and vascular endothelial growth factor (VEGF)-C, and VEGF-D were measured in plasma and detected by Millipore Technology (Merck, Kenilworth, NJ, USA). Values below detection limit were set to half of the minimal detected value.

### Immunoglobulins in plasma from immunized mice

IgG1 and IgG2c against PADRE (Biopeptide, San Diego, CA, USA) and against collagen α1α2(IV) peptides without PADRE (KJ Ross-Petersen Aps, Copenhagen, Denmark) were measured using ELISA. Microtiter 96-well plates were coated with 10 µg/mL of peptides and incubated at 4 °C overnight. The wells were blocked with 2% bovine serum albumin (BSA) (Sigma-Aldrich, St Louis, MO, USA) in PBS for 2 h at room temperature (RT) followed by an addition of individual mouse plasma samples (dilution 1:500 for IgG2c, 1:5000 for IgG1 in 0.2% BSA-PBS) overnight at 4 °C. Bound antibodies were detected with alkaline phosphatase-conjugated rat anti-mouse IgG1 (clone X56, BD Pharmingen, San Jose, CA, USA) or alkaline phosphatase- conjugated rat anti-mouse IgG2c (Southern Biotech, Birmingham, AL, USA) for 2 hours at RT. Finally, wells were incubated with alkaline phosphatase substrate (Thermo Fisher Scientific, Hampton, NH, USA) before absorbance was measured at 405 nm. Washings were performed with PBS containing 0.05% Tween 20. Antibody specificity for IgG1 was determined by a soluble-phase competitive ELISA. Different concentrations of mouse collagen type IV protein (Corning, MA, USA), Cu-oxidized LDL, or bovine serum albumin (BSA) (Sigma-Aldrich, St Louis, MO, USA) were added to mouse plasma pooled from each treatment and incubated overnight at 4 °C, before addition to microtiter plates coated with collagen α1α2(IV)-PADRE peptides (Biopeptide, San Diego, CA, USA) and detection with alkaline phosphatase-conjugated rat anti-mouse IgG1 as described above. IgG1 against oxidized LDL were measured essentially as described above. Plates were coated with 10 µg/ml Cu-oxidized LDL, and plasma from immunized were diluted 1:10.

### Plasma cholesterol and triglyceride levels

Total cholesterol and triglyceride levels were measured in plasma and analyzed colorimetrically by Infinity^TM^ Cholesterol and Triglyceride kits (Thermo Scientific, Waltham, MA, USA) according to the manufacturer’s instructions.

### Oil Red O staining of en face preparations of aorta

*En face* preparations of aortas were fixed in Histochoice (Amresco, Solon, OH, USA) overnight at 4 °C before Oil Red O staining. Glasses were dipped in 78% methanol, followed by 40 min staining in a solution containing 70 mL of 0.2% Oil Red O (Sigma-Aldrich, St Louis, MO, USA) in methanol and 20 mL of 1 M NaOH. The glasses were washed twice in 78% methanol and then washed twice in distilled water. Glasses were mounted with Aqua Pertex (Histolab Products AB, Askin, Sweden).

### Immunohistochemistry and histology of subvalvular sections and brachiocephalic artery

For analysis of the aortic root subvalvular plaques, hearts were embedded in OCT (Optimum Cutting Temperature) Tissue-Tek (Sakura, Leiden, Netherlands) and 10 µm thick sections were cut from the aortic root. Sections were fixed in Histochoice (Amresco, Solon, OH, USA) for 20 min followed by acetone for another 10 min. Before permeabilization of the cells with 0.5% Triton X-100 (Sigma-Aldrich, St Louis, MO, USA) for 10 min, the sections were washed twice with PBS. After a second wash, the slides were incubated with 3% H_2_O_2_ in methanol prior blocking with 10% goat serum for 1 h. Avidin/biotin blocking kit (Vector Laboratories, Burlingame, CA, USA) was used to reduce background. The slides were incubated for 1 hour with rabbit polyclonal CD68 antibody (Abcam, Cambridge, UK), rat monoclonal mouse CD4 antibody (BD Biosciences), goat polyclonal collagen IV alpha 1 antibody (Novusbio, UK), rabbit monoclonal smooth muscle alpha actin antibody (Abcam, Cambridge, UK), or isotype control monoclonal rabbit IgG (Abcam, Cambridge, UK). Biotinylated Goat Anti-Rabbit (Vector Laboratories, Burlingame, CA, USA) was used as secondary antibody. Staining was developed using ABC-elite DAB detection kit, according to manufacturer’s instructions (Vector Laboratories, Burlingame, CA, USA) and counterstained with Mayer’s Hematoxylin (Histolab Products AB, Askim, Sweden). Collagen was visualized using van Gieson solution (Sigma-Aldrich, Stockholm, Sweden) for 1.5 minutes before washing in water and 95% ethanol. Oil Red O stainings were performed by fixation of sections in Histochoice, staining with 0.4% Oil Red O (Sigma-Aldrich, St Louis, MO, USA) in isopropanol for 10 min and washed in water before nuclei-staining with Harris’ hematoxylin (Histolab Products AB, Askim, Sweden). Brachiocephalic arteries were embedded in paraffin and cut in 5 µm sections. Cross-sections were stained with Mayer’s hematoxylin (Histolab Products AB, Askim, Sweden) for 4 min, washed in water and then stained with 1% erythrosine. Necrotic core area was determined as the acellular area. Stained sections were scanned and digitalized using an Aperio ScanScope digital slide scanner (Scanscope Console v8.2.0.1263, AmperioTechnologies, Inc., Vista, CA, USA) and positively stained areas were quantified using Biopix iQ software (BioPixAB, Gothemborg, Sweden) by a blinded observer.

### Quantitative PCR of IFN-g and IL-4 in brachiocephalic artery

Brachiocephalic arteries were lysed in 1 mL Trizole and homogenized before addition of 200 µL chloroform and incubation for 15 min. Samples were then centrifuged at 12000 g at 4 °C for 15 min to reach phase separation. The aqueous phase was retrieved and RNA was precipitated by addition of 2 µL of linear polyacrylamide (Invitrogen, Carlsbad, CA, USA) and 500 µL of isopropanol at −20 °C overnight followed by centrifugation at 12000 g at 4 °C for 15 min, before it was washed in 75% ethanol. RNA was dissolved in RNAse free water and concentration was measured by NanoDrop spectrophotometer. cDNA was synthesized from 600 ng of RNA using high capacity RNA to cDNA kit (Applied Biosystems, Stockholm, Sweden). Realtime quantitative PCR was performed using Taqman Fast Advanced Master mix (Applied Biosystems, Stockholm, Sweden) with IL4 (Mm00445259_m1) or INFγ (Mm01168134_m1) gene expression assays from TaqMan (ThermoFisher Scientific). Results were calculated using ΔΔCT method. All results are shown as mean relative expression to control PBS-treated mice and controlled endogenously to the housekeeping genes β-actin (Mm00607939_s1) and GAPDH (Mm99999915_g1). IL-4 expression was below detection limit in the majority of samples.

### Statistics

Statistical analysis was performed using GraphPad Prism (version 7) (GraphPad Software, La Jolla, CA, USA). Differences between multiple groups were tested with one-way ANOVA followed by Sidak’s multiple comparisons test for parametric data, while Kruskal-Wallis test with Dunn’s multiple comparison test was used in non-parametric data. D’Agostino-Pearson omnibus normality test was used to test for normality distribution. When testing two different groups, Mann Whitney or Student’s t-test for normalized values was used, according to the distribution of the variables in question. GraphPad outlier test was performed to identify outliers. All the values are represented as median (interquartile range) or mean ± standard deviation as specified. P-value < 0.05 was considered significant.

## Results

We have recently demonstrated an association between autoantibodies against MDA-modified collagen type IV and risk for development of myocardial infarction^8^. To investigate if immune responses against collagen type IV could contribute to vascular injury affecting the development of atherosclerosis we immunized ApoE^−/−^ mice with native or MDA-collagen type IV using Alum, Freund’s incomplete or Freund’s complete as adjuvants. However, in neither case a consistent antibody response was induced in the mice. Previous studies have shown the coupling of peptides to PADRE induces a strong antibody response against the peptides^20,21,25^. As an alternative approach to enhance the antibody response we coupled collagen type IV α1 and α2 peptides, previously shown to result in antibodies against collagen type IV^23^, to PADRE. Mice were immunized with either a low or a high dose, 25 or 50 µg respectively, of collagen α1α2(IV)-PADRE, and control mice were immunized with the corresponding amount of PADRE (8 or 16 µg). An additional group of mice received PBS alone to control for possible effects of PADRE. Alum was used as adjuvant in all mice. In a first step, we analyzed the response of immunization of PADRE alone compared to PBS control mice. In the second step, we studied the effect of collagen α1α2(IV)-PADRE peptide immunization on atherosclerotic lesion formation.

### Immunization of ApoE^−/−^ mice with PADRE results in IgG1 antibodies

**Figure 1:**
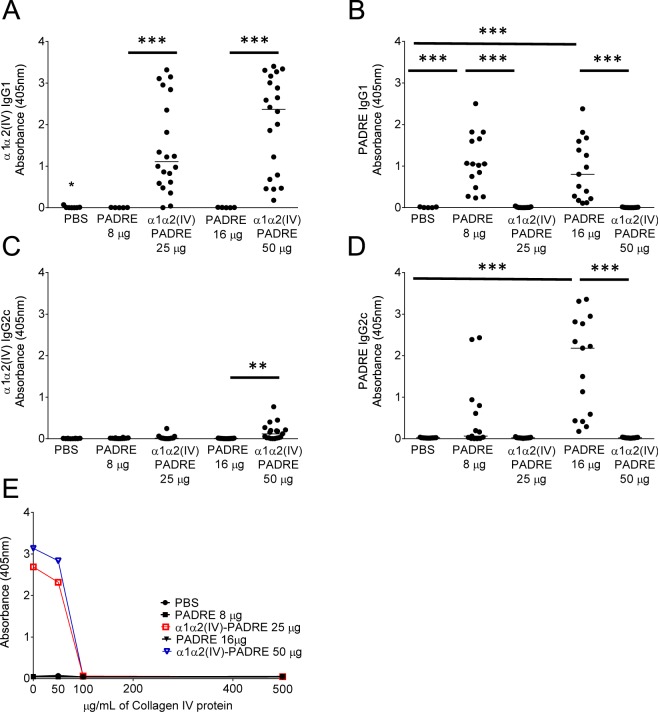
Immunization of ApoE^−/−^ mice with collagen α1α2(IV)-PADRE results in an IgG1 response against collagen type IV. ApoE^−/−^ mice were immunized with collagen α1α2(IV)-PADRE peptides (25 or 50 µg), PADRE (8 or 16 µg) or PBS. Plasma from immunized mice were analyzed for IgG1 (dilution 1:5000) or IgG2c (dilution 1:500) for binding to en α1α2(IV) peptides (**A**,**C**) or PADRE (**B**,**D**) by ELISA. The specificity of IgG1 binding to collagen α1α2(IV) peptides was analyzed by addition of mouse collagen type IV protein at different concentrations (**E**). Kruskal-Wallis test followed by Dunn’s multiple comparisons post-test. Each dot represents one mouse and the bar denotes median value. (**E**) Values are represented as means of triplicates ± SD. **p < 0.005, ***p < 0.0001.

Plasma from mice injected with either 8 or 16 µg of PADRE presented a strong IgG1 (Th2) response against PADRE (1.1 (0.5–1.7) AU vs 0 (0–0) AU, p < 0.0001 (8 µg PADRE vs PBS) and (0.8 (0.2–1.6) AU vs 0 (0–0) AU, p < 0.0001 (16 µg PADRE vs PBS)) (Fig. 1B), whereas plasma from mice injected with PBS did not contain antibodies against PADRE. In addition, a less strong IgG2c response (Th1) were present in mice immunized with a high dose of PADRE (2.2 (0.4–2.8) AU vs 0 (0–0) AU, p < 0.0001 (16 µg PADRE vs PBS)) (Fig. 1D).

### T cell characterization in ApoE^−/−^ mice immunized with PADRE

**Figure 2:**
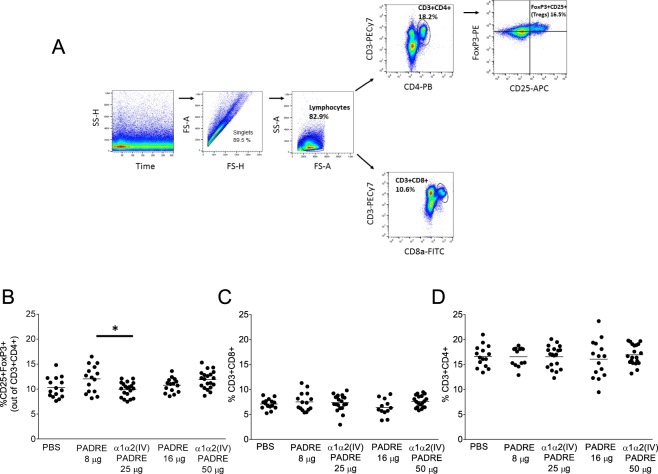
Characterization of T cells in spleen of ApoE^−/−^ mice immunized with collagen α1α2(IV)-PADRE. Splenocytes from mice immunized with collagen α1α2(IV)-PADRE peptides, PADRE or PBS were analyzed with flow cytometry. Representative flow cytometry gating strategy for CD3+CD4+, CD3+CD8+ and CD25+ FoxP3+. (**A**) Percentage of CD25+FoxP3+ (Treg) out of CD3+CD4+, (**B**) CD3+CD8+, (**C**) and CD3+CD4+cells, (**D**) for each treatment. ANOVA followed by Sidak’s multiple comparisons post-test with the bar denoting mean value. Each dot represents one mouse. *p < 0.05.

**Figure 3:**
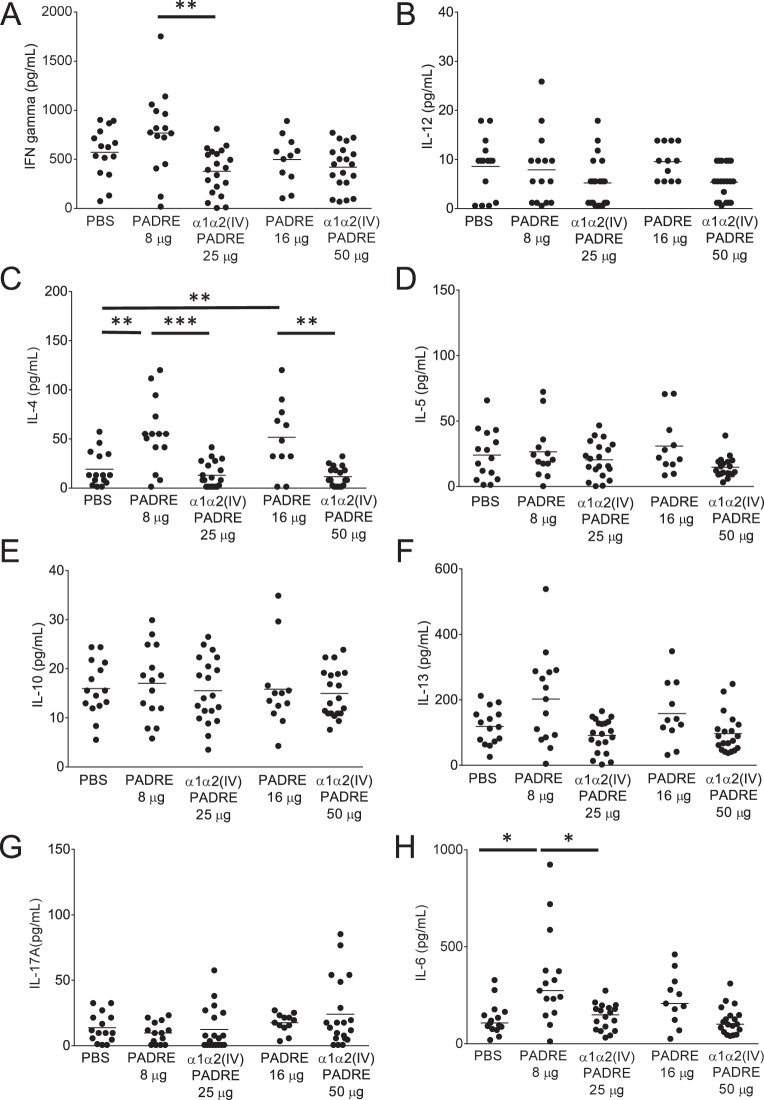
T cell related cytokines released by splenocytes from ApoE^−/−^ immunized with collagen α1α2(IV)-PADRE. Splenocytes from mice immunized with collagen α1α2(IV)-PADRE peptides, PADRE or PBS were stimulated with anti-CD3/CD28 beads and IFN-g (**A**), IL-12 (**B**), IL-4 (**C**), IL-5 (**D**), IL-10 (**E**), IL-13 (**F**), IL-17A (**G**) and IL-6 (**H**) were analyzed. ANOVA followed by Sidak’s multiple comparisons post-test where the bar denotes mean for normally distributed variables, or Kruskal-Wallis test followed by Dunn’s multiple comparisons post-test where the bar shows median value for non-normally distributed variables. Each dot represents one mouse. *p < 0.05, **p < 0.005, ***p < 0.0001.

We then determined if PADRE-immunization altered T cell populations in ApoE^−/−^ mice. Representative gating strategy for the selection of CD3^+^CD4^+^, CD3^+^CD8^+^ and regulatory T cells expressing CD25^+^FoxP3^+^ from total splenocytes analyzed by flow cytometry are shown in Fig. 2A. No significant differences regarding either of the T cell populations in PADRE versus PBS-immunized mice were found (Fig. 2). In order to assess the pro- (Th1) versus anti- (Th2) inflammatory cytokine patterns, supernatants from anti-CD3/CD28-stimulated splenocytes and plasma from the immunized mice were analyzed. Immunization with PADRE resulted in an increase in IL-4 levels in splenocyte cultures compared to PBS-immunized mice (55.4 ± 3.6 pg/ml vs 19.1 ± 17.7 pg/ml, p = 0.0004 (8 µg PADRE vs PBS) and 51.6 ± 36.6 pg/ml vs 19.1 ± 17.7 pg/ml, p = 0.004 (16 µg PADRE vs PBS)) (Fig. 3C), whereas no differences in IFN-γ, IL-12, IL-5, IL-10, IL-13 or IL-17A were present (Fig. 3). Th1 or Th2 cytokines levels in plasma were below detection limit, except for IL-5 (Supplementary Fig. 3A), which did not differ between the groups. In addition, PADRE immunization resulted in an increase in IL-6 levels assessed in splenocyte cultures (274 (160–377) pg/ml vs 106 (75–164) pg/ml, p = 0.008 (8 µg PADRE vs PBS)) (Fig. 3H) and in plasma (37.6 (6.5–86.0) pg/ml vs 0.4 (0.4–1.5) pg/ml, p < 0.0001 (8 µg PADRE vs PBS) and (23.7 (14.4–106) pg/ml vs 0.4 (0.4–1.5) pg/ml, p < 0.0001 (16 µg PADRE vs PBS)) (Supplementary Fig. 3B).

### Immunization of ApoE^−/−^ mice with PADRE decrease SMC in plaques

**Figure 4:**
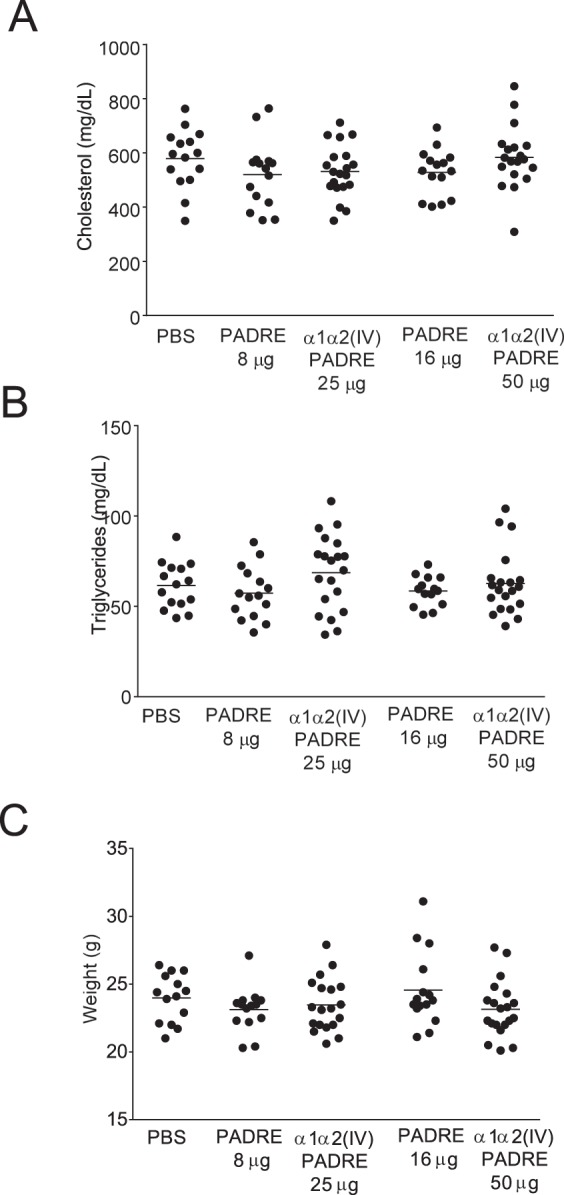
Immunization of ApoE^−/−^ mice with collagen α1α2(IV)-PADRE does not alter plasma lipid levels or mouse weight. Plasma cholesterol (**A**), triglycerides (**B**) and body weight (**C**) were measured in ApoE^−/−^ immunized with collagen α1α2(IV)-PADRE peptides, PADRE or PBS. Groups were compared using ANOVA. Each dot represents one mouse. The bar denotes mean values.

**Figure 5:**
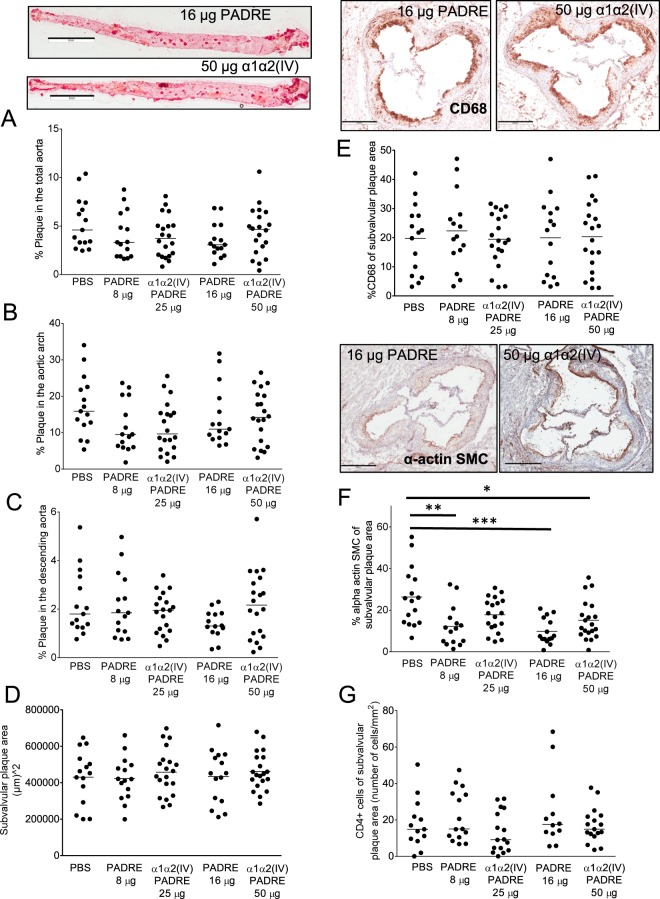
Immunization of ApoE^−/−^ mice with collagen α1α2(IV)-PADRE does not alter atherosclerotic plaque size. ApoE^−/−^ mice were immunized with collagen α1α2(IV)-PADRE peptides (25 or 50 µg), PADRE (8 or 16 µg) or PBS. Plaque area was analyzed in *en face* preparations of total aorta (**A**), aortic arch (**B**) and descending aorta (**C**) stained with Oil red O (scale bars = 5 mm). Subvalvular plaque areas (**D**) were measured in cross-sections from the aortic root. Macrophage (anti-CD68) (**E**), smooth muscle cell (alpha-actin SMC) (**F**), and CD4 cell content were analyzed in subvalvular plaques. Representative photomicrographs are shown for immunizations with 50 µg of collagen α1α2(IV) and 16 µg of PADRE (scale bars = 400 µm). ANOVA followed by Sidak’s multiple comparisons post-test. Each dot represents one mouse and the bar denotes mean values. *p < 0.05, **p < 0.005.

Immunization of ApoE^−/−^ mice with PADRE did not affect mouse weight, or plasma cholesterol and triglyceride levels (Fig. 4). Next, we asked whether the immunization of PADRE affect plaque size. However, neither total plaque area in the aortic arch or descending aorta nor the subvalvular or the brachiocephalic artery plaque areas were altered upon immunization with PADRE (Fig. 5A–D, Supplementary Fig. 4D). Plaque content of CD68 cells, collagen, collagen IV or CD4 cells were not significantly different in PADRE immunized mice compared to PBS control mice (Fig. 5E,G, Supplementary Fig. 4B,C). However, the SMC content in subvalvular lesions were decreased in PADRE immunized mice (12.2 ± 9.6% vs 26.3 ± 14.2%, p = 0.001 (8 µg PADRE vs PBS) and 9.8 ± 6.2% vs 26.3 ± 14.2%, p = 0.0002 (16 µg PADRE vs PBS)) (Fig. 5F). Furthermore, lipid content in plaques from mice immunized with the high concentration of PADRE was decreased (18.1 ± 2.7% vs 27.1 ± 5.9%, p < 0.0001 (16 µg PADRE vs PBS) (Supplementary Fig. 4A).

Immunization with Alum has previously been shown to have a protective effect on atherosclerosis, suggested to result from an enhanced immune reaction against oxidized LDL at the injection site^26^. To investigate if PADRE could enhance these immune responses, we analyzed antibodies against Cu-oxidized LDL in plasma from immunized mice. However, only very low antibody amounts were present, and no difference between PADRE immunized mice and PBS control mice was observed (Supplementary Fig. 1).

### Immunization of ApoE^−/−^ mice with collagen type IV α1α2 peptides result in IgG1 antibodies against collagen type IV

In the next step we analyzed the effect of an immune response against collagen IV on atherosclerotic disease. Immunization with either 25 or 50 µg of collagen α1α2(IV)-PADRE induced a strong IgG1 (Th2) response against collagen type IV α1α2 peptides, although the response varied within mice from the same group (Fig. 1A). A less strong IgG2c (Th1) response was present in mice immunized with 50 µg α1α2(IV)-PADRE (Fig. 1C). In contrast to PADRE immunized mice, antibodies from collagen α1α2(IV)-PADRE immunized mice did not bind to PADRE alone (Fig. 1B,D). Plasma from mice injected with PBS or PADRE did not contain antibodies against collagen α1α2(IV) peptides. Furthermore, neither IgG1 nor IgG2c antibodies against collagen type IV were present in plasma from ApoE^−/−^ mice before immunization (data not shown). To determine if induced antibodies against collagen peptides recognized the collagen type IV protein, we tested whether IgG1 binding to collagen α1α2(IV) peptides could be competed by addition of the collagen type IV protein. Indeed, pre-incubation of plasma with collagen type IV at a concentration of 100 µg/mL totally blocked binding to collagen peptides (Fig. 1E), whereas pre-incubation with oxidized LDL or BSA had no effect (Supplementary Fig. 2).

In the following analyses collagen α1α2(IV)-PADRE immunized ApoE^−/−^ mice were compared to mice immunized with the corresponding amount of PADRE. Since PADRE immunization alone induced antibodies against PADRE, which the collagen-PADRE conjugate did not, we also compared collagen α1α2(IV)-PADRE immunized mice with PBS control mice.

### T cell characterization in ApoE^−/−^ mice immunized with collagen type IV α1α2 peptides

Immunization with collagen α1α2(IV)-PADRE peptides did not affect CD4^+^ or CD8^+^ T cells compared to control groups (Fig. 2C,D). Mice immunized with 25 μg of collagen α1α2(IV)-PADRE displayed decreased percentages of regulatory T cells compared to 8 μg of PADRE, whereas no difference was detected in mice immunized with the higher concentration of peptides (Fig. 2B). Th1 associated IFN-γ and the inflammatory cytokine IL-6 were decreased in splenocytes cultures from mice immunized with collagen α1α2(IV)-PADRE mice compared to 8 μg of PADRE (Fig. 3A,H). This effect was not observed for the higher dose. Supernatants from collagen α1α2(IV)-PADRE immunized mice also displayed decreased levels of Th2-associated IL-4 (Fig. 3C) compared to PADRE immunized mice, but not compared to PBS control mice. Furthermore, IL-6 levels in plasma were decreased in α1α2(IV)-PADRE immunized mice compared to PADRE immunized mice, but not to PBS mice (Supplementary Fig. 3). No significant differences were noticed for IL-12, IL-5, IL-10, IL-13, or IL-17A from splenocytes (Fig. 3B,D–G) or IL-5 in plasma (Supplementary Fig. 3A). IFN-γ, IL-12, IL-10, IL-13, IL-4 and IL-17A levels in plasma were below detection limit.

### Immunization of ApoE^−/−^ mice with collagen type IV α1α2 peptides does not affect atherosclerotic plaque size

Immunization of ApoE^−/−^ mice with collagen α1α2(IV)-PADRE did not affect mouse weight, plasma cholesterol or triglyceride levels (Fig. 4A–C). Next, we investigated the effect of an immune response directed against collagen type IV on atherosclerotic plaque formation. First, we analyzed plaque areas by Oil Red O staining of *en face* preparations of the aorta. However, neither percentage of plaque in total aorta, nor in the aortic arch or in the descending aorta were affected by immunization with collagen α1α2(IV)-PADRE (Fig. 5A–C). We then measured subvalvular plaque area in the aortic root, a location known to be one of the first places for plaque formation in mice^27^ as well as in the brachiocephalic artery. Again, no differences between collagen α1α2(IV)-PADRE and PADRE immunized groups (Fig. 5D, Supplementary Fig. 4D) were observed. To evaluate if antibodies against collagen α1α2(IV) influence plaque inflammation, macrophage content and CD4 cells in subvalvular plaques were analyzed, but no differences were found between the groups (Fig. 5E,G). Furthermore, plaque smooth muscle cell content was measured. The levels of alpha actin smooth muscle cells were increased in the high concentration of collagen α1α2(IV)-PADRE immunized mice compared to PBS mice (Fig. 5F). Since an even stronger effect was seen in PADRE immunized mice, this effect appears to be mainly due to the presence of PADRE. Lipid content was increased in mice immunized with the high dose of collagen α1α2(IV)-PADRE compared to PADRE control mice, but no difference was present compared to PBS mice or in mice injected with the lower dose of antigen. Collagen or collagen type IV content in the subvalvular plaques did not differ between the groups (Supplementary Fig. 4A–C). We did not observe any difference in necrotic core area of plaques from the brachiocephalic artery or in IFN-γ expression at the same location (Supplementary Fig. 4E,F). IL-4 expression was below detection limit.

**Figure 6:**
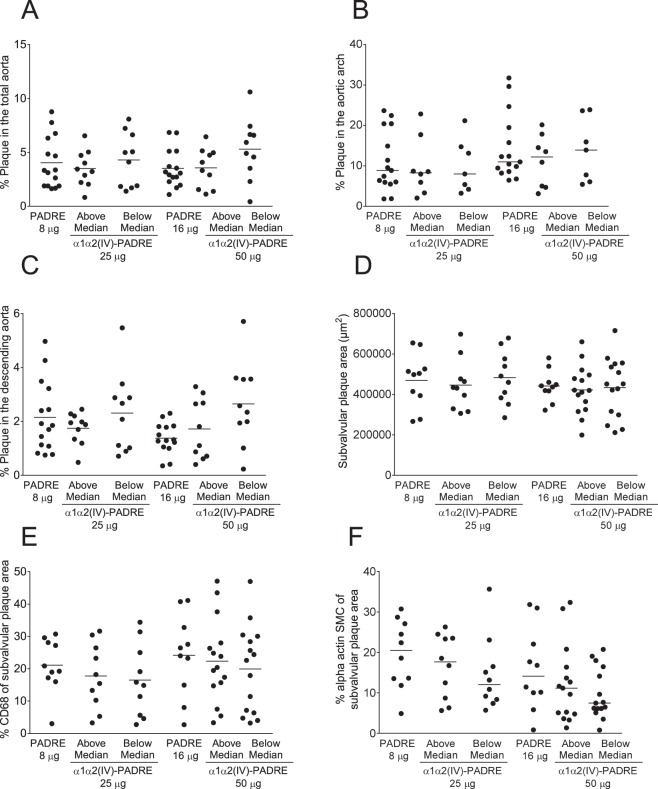
IgG1 levels in ApoE^−/−^ mice immunized with collagen α1α2(IV)-PADRE do not influence atherosclerotic plaque size, macrophage or SMC content. Levels of IgG1 against collagen α1α2(IV) peptides in ApoE^−/−^ mice immunized with α1α2(IV)-PADRE were divided in above and below median and analzyed for percentage of plaque in total aorta (**A**), aortic arch (**B**), descending aorta (**C**), subvalvular plaque area (**D**), CD68 (**E**) and smooth muscle cell alpha actin (**D**). Groups were compared using ANOVA or Kruskal-Wallis test. Each dot represents one mouse and the bar denotes mean or median values.

To determine if the levels of induced collagen α1α2(IV) antibodies were affecting plaque size and plaque content, we divided collagen α1α2(IV)-PADRE mice in two groups: below and above median values of IgG1 collagen α1α2(IV) and compared to PADRE immunized mice. However, neither plaque areas nor macrophage or SMC content, were significantly associated to antibody levels (Fig. 6A–F).

### Immunization of ApoE^−/−^ mice with collagen type IV α1α2 peptides does not affect endothelial growth factors in plasma

**Figure 7:**
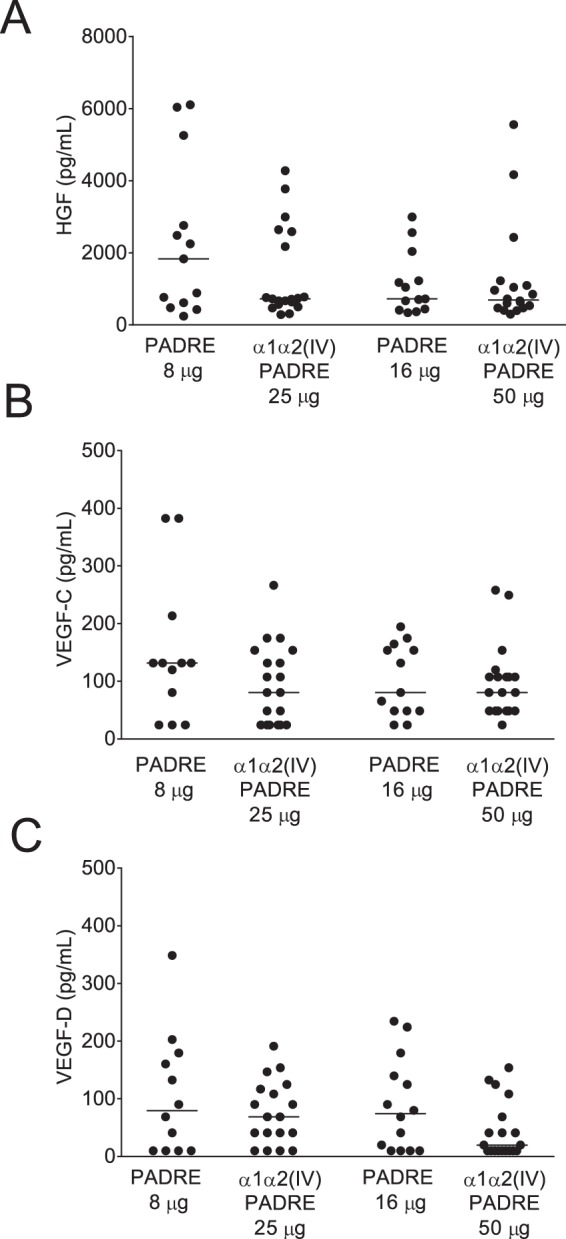
Immunization of ApoE^−/−^ mice with collagen α1α2(IV)-PADRE does not affect endothelial growth factors. HFG (**A**), VEGF-B (**B**) and VEGF-D (**C**) were measured in plasma from ApoE^−/−^ mice immunized with collagen α1α2(IV)-PADRE or PADRE by multiplex technology. Mann-Whitney test or t-test was used with bars showing median or mean value, respectively. Each dot represents one mouse.

Finally, we investigated whether antibodies against collagen type IV peptides resulted in endothelial stress by measuring the endothelial growth factors HGF, VEGF-C and -D in plasma of immunized mice. No significant differences between collagen α1α2(IV)-PADRE and PADRE groups were detected (Fig. 7A–C).

## Discussion

Collagen type IV is one of the major components of basement membranes underlying endothelial cells and surrounding smooth muscle cells^28^. Observational studies have identified associations between autoantibodies against collagen type IV and risk for development of cardiovascular disease^8,29^. In the present paper we investigated the effect on an immune response against the basement membrane collagen type IV on atherosclerotic disease. To achieve this, atherosclerotic ApoE^−/−^ mice were immunized with peptides from collagen type IV α1 and α2 chains, the most common α-chains, present in vascular basement membranes and in human atherosclerotic plaques^17^ as well as in murine tissues rich in basement membranes including heart^30^. Collagen type IV peptides, located at the C-terminal non-collagenous (NC1) domain of the collagen, were chosen based on previous studies showing that immunization with these peptides resulted in antibodies specific for collagen α1(IV) and α2(IV) chains and also had the ability to stain cryostat mouse and rabbit sections^23^. The NC1 domain is important for the assembly of collagen type IV and it is involved in anti-angiogenic activity and in inhibition of endothelial cell proliferation^31^. In the present study, collagen type IV peptides were coupled to PADRE to enhance an antibody response. By this approach we were able to induce a strong IgG1 response against collagen type IV peptides. The antibodies recognized collagen type IV protein, as confirmed by the competition assay. However, assessing atherosclerotic plaque development at four different locations (aortic root, aortic arch, descending aorta, and brachiocephalic artery) we found no differences in the atherosclerotic plaque area between collagen α1α2(IV)-PADRE immunized mice and PADRE immunized or PBS control mice. Additionally, collagen α1α2(IV)-PADRE antibody levels, divided in below and above median, were not associated to plaque size at the different locations. Finally, the immunization with collagen type IV peptides did not influence plaque composition regarding CD68 cells, collagen, or CD4 cells. Although we noted an increase in SMC in collagen α1α2(IV)-PADRE immunized mice compared to PBS mice, this effect was also present in PADRE immunized mice, arguing for that the effect is mainly due to the presence of PADRE. Taken together, these data indicate that antibodies against collagen type IV α1α2 do not influence atherosclerotic plaque formation in ApoE^−/−^ mice.

Even though our hypothesis was discarded, we obtained some interesting findings to be discussed. Surprisingly, PADRE immunized control mice developed a strong Th2 antibody response against PADRE, whereas collagen α1α2(IV)-PADRE immunized mice only developed a Th2 antibody response against collagen α1α2(IV), despite PADRE being present in the immunized peptides. This suggests that conjugation of PADRE to peptides alters its immunogenicity, possibly due to less exposure of the coupled carboxyterminal of PADRE. Furthermore, splenocytes from PADRE immunized mice secreted enhanced levels of the Th2 cytokine IL-4 compared to both collagen α1α2(IV)-PADRE immunized mice and PBS control mice, indicating that PADRE alone results in a stronger Th2 response than if conjugated. In addition, PADRE immunization resulted in an increase in IL-6 levels in plasma, which the conjugate did not, further indicating that conjugation of PADRE diminishes its immune-stimulatory effects.

In atherosclerosis, CD4^+^ Th2 immune responses are considered to be anti-inflammatory due to the secretion and action of IL-4 or IL-5, linked to B cell activation and antibody production^32^. In the present study, a decrease in smooth muscle cell content was present in PADRE immunized mice, suggesting increased plaque vulnerability. It is possible that the increase in IL-4 may affect SMC migration, proliferation or apoptosis in the lesions. IL-4 deficiency in atherosclerotic mice results in reduced plaque formation, however whether IL-4-deficiency has an effect on plaque SMC content was not studied in those papers^33–36^. Interestingly, previous *in vitro* studies of smooth muscle cells from human umbilical artery or aorta showed decreased proliferation upon IL-4 stimulation^37,38^, suggesting that the increase in IL-4 seen in PADRE immunized mice may inhibit SMC proliferation in the plaques, resulting in a reduced SMC content. However, since collagen α1α2(IV)-PADRE immunized mice also displayed reduced SMC content compared to PBS control mice without a concomitant increase in IL-4, it is likely that additional mechanisms are of importance. In this context it is interesting that both PADRE and collagen α1α2(IV)-PADRE immunized mice induced strong antibody responses. It is possible that immune complexes binding to e.g. Fc receptors on smooth muscle cells^39,40^, or other plaque cells affecting smooth muscle cells, could explain the reduced smooth muscle cell content observed in these groups.

Although no differences in plaque size or plaque content were present in collagen α1α2(IV)-PADRE immunized mice, we cannot exclude that the formed antibodies did not have an effect on other atherosclerotic processes. It is also possible that antibodies directed against other collagen type IV sites could influence atherosclerotic plaque formation in ApoE^−/−^ mice. Collagen type IV protein is located in basement membranes and modification of basement membrane components due to lipid oxidation may result in decreased endothelial cell attachment, which may in turn promote cardiovascular events^41,42^. Interestingly, it has been proposed that rheumatic fever may be associated with an autoantibody response to collagen type IV affecting the endothelial-cell layer throughout the body^43^. Mutations in collagen α1α2(IV) in mice lead to hemorrhages during development suggesting its crucial role in vasculature^44^. Studies on Col4a1^+/Raw^ mice present a defective deposition of collagen IV in the descending aorta, affecting endothelial cell function^45^. Thus it is possible that collagen α1α2(IV) autoantibodies formed in the present study could have an effect on endothelial cells. In the current study, however, endothelial growth factors (HGF, VEGF-C and –D) in plasma did not differ between the groups. It is also possible that antibodies against collagen type IV could have an effect on early plaque formation, and that the lesions in these mice were too advanced to detect differences between the groups. However, since we found no differences in plaque formation at any of the three different locations in the aorta or in the brachiocephalic artery, known to develop atherosclerotic plaques at different time points and vary in plaque progression, this is less likely.

The lack of effect on atherosclerosis by immunization with collagen type IV α1α2 peptides suggests that autoantibodies against collagen type IV, previously associated with cardiovascular disease in humans^8,29^, do not play a pathogenic role. In a previous study immunization against aldehyde-modified laminin, another extracellular matrix protein present in basement proteins, resulted in increased atherosclerosis in apoE^−/−^ mice^2^. Among the many possible reasons for the discrepancy between the studies, could be the different specificity of the antibodies, which in the present study only recognized the native protein, instead of the aldehyde-modified form. It is also important to recognize differences in human versus murine atherosclerosis when extrapolating results between the species. The earliest vascular change in human vessels is an intimal thickening of the vessel, consisting of a layer of SMCs and extracellular matrix. This is followed by an accumulation of lipoproteins and macrophages^46^. In mice, the opposite occurs, starting with lipid and macrophage accumulation, which are followed by SMC migration and fibrous cap formation. Thus, even though an immune reaction against the collagen type IV does not affect atherosclerosis in mice, we cannot rule out that autoantibodies in human disease still may play a functional role.

In summary, ApoE-deficient mice immunized with collagen α1α2(IV)-PADRE responded with a strong Th2 immune response, measured by IgG1 antibody levels, but did not have an effect on atherosclerosis.

## Supplementary information

Supplementary figure 1-4
