# Supplementary material for: Activation of immune responses against the basement membrane component collagen type IV does not affect the development of atherosclerosis in ApoE-deficient mice
Source: Sci Rep. 2019 Apr 12;9:5964. doi: 10.1038/s41598-019-42375-8 (PMC6461614; doi:10.1038/s41598-019-42375-8)
Supplement: Supplementary file 1 — Supplementary figure 1-4 [file 41598_2019_42375_MOESM1_ESM.pdf]

Supplementary information

Activation of immune responses against the basement membrane component collagen type IV does not affect the development of atherosclerosis in ApoE-deficient mice

Vallejo J, Dunér P, To F, Engelbertsen D, Gonçalves I, Nilsson J, Bengtsson E.

Department of Clinical Sciences Malmö, Skåne University Hospital, Lund University, Malmö, Sweden.

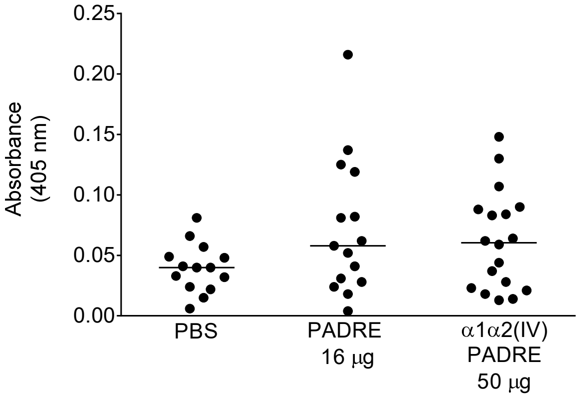

Supplementary figure 1: Antibodies against oxidized LDL in PADRE and collagen α1α2(IV)-PADRE immunized mice.

IgG1 antibodies against Cu-oxidized LDL in plasma (dilution 1:10) from mice immunized with collagen α1α2(IV)-PADRE peptides, PADRE or PBS were analyzed by ELISA. Kruskal-Wallis test. Bar shows median value. Each dot represents one mouse.

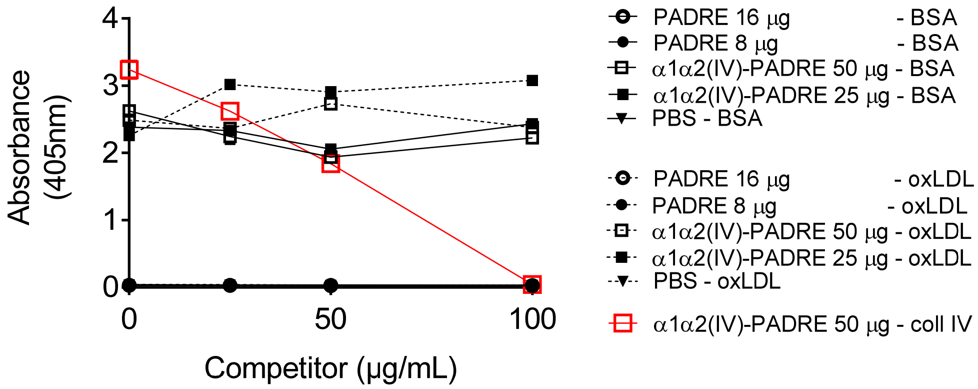

Supplementary figure 2: Specificity of antibodies to collagen type IV in collagen α1α2(IV)-PADRE immunized mice.
ApoE$^{-/-}$ mice were immunized with collagen α1α2(IV)-PADRE peptides (25 or 50 µg), PADRE (8 or 16 µg) or PBS. The specificity of IgG1 binding to collagen α1α2(IV) peptides was analyzed by preincubation with Cu-oxidized LDL (dashed line), bovine serum albumin (black line), or mouse collagen IV protein (red line) at different concentrations. Values are presented as means of triplicates ± SD.

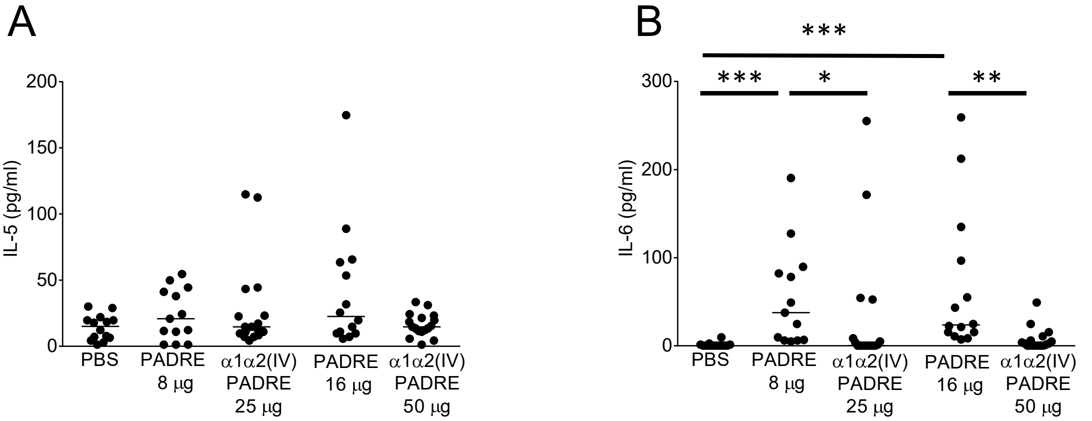

Supplementary figure 3: Plasma IL-5 and IL-6 levels in ApoE$^{-/-}$ immunized with collagen α1α2(IV)-PADRE.

Plasma from mice immunized with collagen α1α2(IV)-PADRE peptides, PADRE or PBS were analyzed for IL-5 (A) and IL-6 (B). Kruskal-Wallis test followed by Dunn's multiple comparisons post-test where the bar shows median value for non-normally distributed variables. Each dot represents one mouse.

* $p<0.05$, ** $p<0.005$

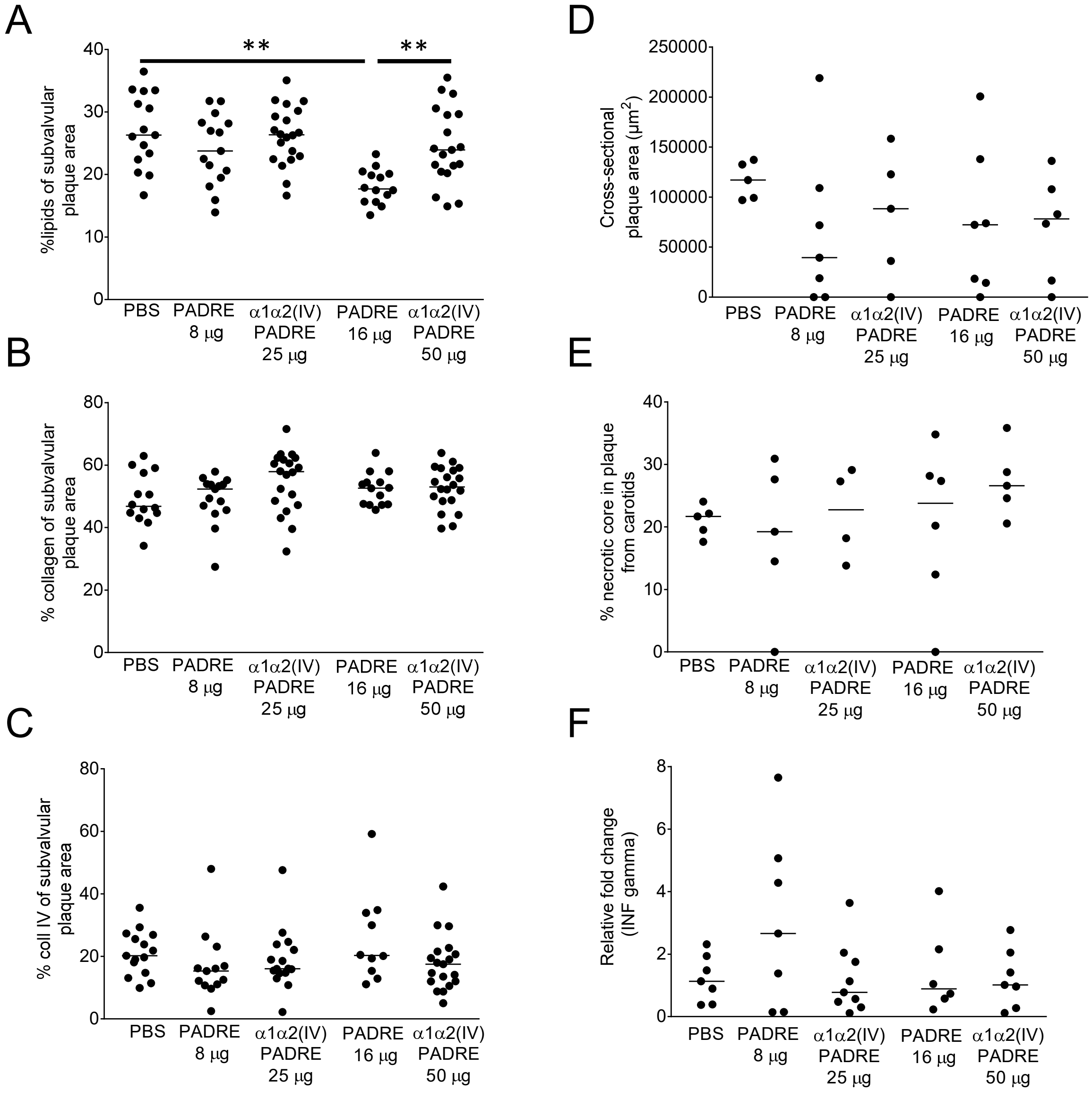

Supplementary figure 4: Analysis of subvalvular and carotid plaques in ApoE$^{-/-}$ mice immunized with collagen α1α2(IV)-PADRE.

ApoE$^{-/-}$ mice were immunized with collagen α1α2(IV)-PADRE peptides (25 or 50 µg), PADRE (8 or 16 µg) or PBS. Lipid (Oil red O) (A), collagen (van Gieson) (B), and collagen type IV (anti-coll IV) (C) content were measured in subvalvular plaques. Cross-sectional plaque area (D) and necrotic core area (E) were analyzed in the brachiocephalic artery. IFNγ mRNA expression in the brachiocephalic artery was analyzed by quantitative PCR (F). ANOVA followed by Sidak's multiple comparisons post-test where the bar denotes mean for normally distributed variables, or Kruskal-Wallis test where the bar shows median value for non-normally distributed variables. Each dot represents one mouse. ** $p<0.005$
